# Downscaling Global Emissions and Its Implications Derived from Climate Model Experiments

**DOI:** 10.1371/journal.pone.0169733

**Published:** 2017-01-11

**Authors:** Shinichiro Fujimori, Manabu Abe, Tsuguki Kinoshita, Tomoko Hasegawa, Hiroaki Kawase, Kazuhide Kushida, Toshihiko Masui, Kazutaka Oka, Hideo Shiogama, Kiyoshi Takahashi, Hiroaki Tatebe, Minoru Yoshikawa

**Affiliations:** 1 Center for Social and Environmental Systems Research, National Institute for Environmental Studies (NIES), 16–2, Onogawa, Tsukuba, Japan; 2 Department of Integrated Climate Change Projection Research, Japan Agency for Marine-Earth Science and Technology (JAMSTEC), 3173–25 Showa-machi, Kanazawa-ku, Yokohama, Japan; 3 College of Agriculture, Ibaraki University, 3-21-1 Chuo, Ami, Japan; 4 Meteorological Research Institute, 1–1, Nagamine, Tsukuba, Japan; 5 Mizuho Information & Research Institute, Inc., 2–3 Kanda-Nishikicho, Chiyoda-ku, Japan; 6 Center for Global Environmental Research, National Institute for Environmental Studies (NIES), 16–2, Onogawa, Tsukuba, Japan; Centro de Investigacion Cientifica y de Educacion Superior de Ensenada Division de Fisica Aplicada, MEXICO

## Abstract

In climate change research, future scenarios of greenhouse gas and air pollutant emissions generated by integrated assessment models (IAMs) are used in climate models (CMs) and earth system models to analyze future interactions and feedback between human activities and climate. However, the spatial resolutions of IAMs and CMs differ. IAMs usually disaggregate the world into 10–30 aggregated regions, whereas CMs require a grid-based spatial resolution. Therefore, downscaling emissions data from IAMs into a finer scale is necessary to input the emissions into CMs. In this study, we examined whether differences in downscaling methods significantly affect climate variables such as temperature and precipitation. We tested two downscaling methods using the same regionally aggregated sulfur emissions scenario obtained from the Asian-Pacific Integrated Model/Computable General Equilibrium (AIM/CGE) model. The downscaled emissions were fed into the Model for Interdisciplinary Research on Climate (MIROC). One of the methods assumed a strong convergence of national emissions intensity (e.g., emissions per gross domestic product), while the other was based on inertia (i.e., the base-year remained unchanged). The emissions intensities in the downscaled spatial emissions generated from the two methods markedly differed, whereas the emissions densities (emissions per area) were similar. We investigated whether the climate change projections of temperature and precipitation would significantly differ between the two methods by applying a field significance test, and found little evidence of a significant difference between the two methods. Moreover, there was no clear evidence of a difference between the climate simulations based on these two downscaling methods.

## Introduction

Climate change studies can be categorized into three main communities. First, climate models (CMs) can be used to simulate climate variables using emission data of greenhouse gases (GHGs) and air pollutants (APs). Second, the impact, adaptation, and vulnerability (IAV) community assesses climate impacts using future climate information and socioeconomic conditions. Third, the integrated assessment model (IAM) community deals with future emissions of GHGs and APs and climate mitigation analyses. Emissions data are necessary for CM projections, and IAMs are used to generate future emissions scenarios that are integrated into CMs. Examples of such scenarios can be found in the Special Report on Emissions Scenarios (SRES) Nakicenovic and Swart [1] and Representative Concentration Pathways (RCPs) [2]. These scenarios are ultimately used in climate model comparison projects such as the Coupled Model Intercomparison Project phase 3 and phase 5 (CMIP3, CMIP5: http://cmip-pcmdi.llnl.gov/cmip5/).

In CM calculations for future scenario analyses, emissions data from IAMs must be disaggregated into a finer scale, or downscaled, for use in CMs. IAMs usually classify the world into 10–30 aggregated regions and simulate energy, land use, and relevant emissions. Regional aggregation provides sufficient detail to capture the characteristics of different parts of the world while avoiding unnecessary complexity. Well-known examples include the Asia Pacific Integrated Model (AIM) [3], Global Change Assessment Model (GCAM) [4], Integrated Model to Assess the Greenhouse Effect (IMAGE) [5], Model for Energy Supply Strategy Alternatives and their General Environmental Impact (MESSAGE) [6], and Regionalized Model of Investments and Technological Development (ReMIND) [7]. However, other research communities, such as the CM and IAV communities, use grid-based spatial resolutions for emissions.

Downscaling methods have been comprehensively reviewed by van Vuuren *et al*. [8], who classified downscaling methods into four categories: algorithmic downscaling, methods of intermediate complexity, fully elaborated models at relatively low levels of aggregation, and fully coupled models at national or grid scales. Downscaling is applicable to various indicators, but few studies have concerned global emissions. The global emissions studies of Hoehne and Ullrich [9] and van Vuuren *et al*. [10] used the algorithmic method. This method can be further classified into proportional and convergence downscaling methods, which have been applied by Hoehne and Ullrich [9] and van Vuuren *et al*. [10], respectively. The former consists of simple proportional downscaling. Meanwhile, the latter consists of allocating population first, and then allocating gross domestic product (GDP) and emissions. Then, the GDP per capita and emissions intensity (i.e., emissions per GDP) for each country are assumed to converge to values that correspond to regional aggregates. Finally, country-level indicators are downscaled to each grid.

Recently, the interaction between IAMs and CMs has received great attention, and several studies have attempted to couple IAMs and CMs [11–15]. van Vuuren *et al*. [16] summarized and classified possible interactions between these types of models. They reviewed relevant research activities and suggested approaches for each research area. The need for such activities will become more urgent after the Shared Socioeconomic Pathways (SSPs) are developed as the next generation of SRES socioeconomic scenarios, because this will allow the climate study communities to interact more dynamically, as discussed by Ebi *et al*. [17] and van Vuuren *et al*. [18]. This research trend should not be limited to the IAM and CM communities, but should also involve the IAV community, as well as air pollution and health studies. Downscaling is a key facet of this broad-scale research activity. However, one question that arises is how downscaling methods affect projected climate variables, such as temperature and precipitation. Answering this question would provide a better understanding of the relationship between IAMs and CMs, and would be valuable to the IAV community.

Motivated by this background, we had three objectives: document the downscaling method adopted in the AIM modeling framework, analyze the characteristics of the downscaled data for future scenarios, and input the downscaled data into the Model for Interdisciplinary Research on Climate (MIROC) to project climate variables with a focus on statistical significance. In the downscaling process, we assumed two extreme cases that sufficiently differentiated the downscaling patterns, thereby enabling the investigation into the effects of the downscaling method on climate projections. We used a downscaling method similar to that described by van Vuuren *et al*. [10] due to its simplicity, clear description, and suitability for long-term scenario assessments.

## Materials and Methods

### General Description

**Fig 1:**
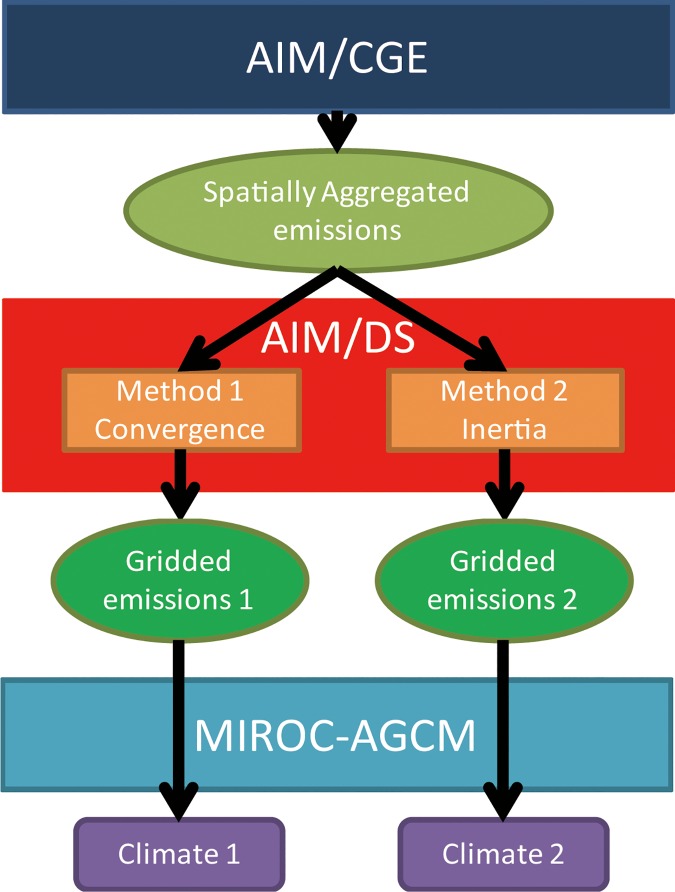
Overall framework (model input and output).

The overall framework of this study (i.e., the model input and output) consisted of three steps (Fig 1). First, AIM/computable general equilibrium (CGE) was used to project emissions of GHGs, and APs were aggregated into 17 regions. Second, AIM/downscaling (DS) was used to downscale the emissions into a 0.5° spatial resolution. Third, MIROC was used to calculate the climate variables using the gridded emissions. We analyzed the results obtained from the AIM/DS and MIROC using two different factors (convergence and inertia) to downscale the emissions. The use of these two factors is explained in more detail in the discussion of the AIM/DS algorithm. We used sulfur emissions to directly compare the different implications of these two methods, as sulfur has a relatively large effect on climate compared to other APs. We differentiated the spatial allocation of sulfur emissions, and used the same information on spatial emissions for the other APs. Because AIM/CGE [19–23] and MIROC [24–26] have already been developed and had many other applications in which documentation is available on the model characteristics. Therefore, we focused predominantly on the AIM/DS methodology and its application.

We analyzed the results from four perspectives. First, we examined the downscaled gridded emissions maps and compared the results for 2005 and 2100 obtained with the two methods. Second, we aggregated the downscaled emission information into national emissions and intensities, and examined the differences between the two methods to explain how the emissions intensity convergence assumption affected the emissions density. Third, we collected grid-based sulfur emission data and assessed the variability and distribution of sulfur emissions. These analyses were performed for each AIM/CGE aggregated region. We calculated the standard deviation (SD) and interquartile range (IQR; i.e., the difference between the upper and lower quartiles) for the gridded information as metrics of the variability of the predictions. Fourth, we applied *t*-tests and field significance tests to the climate model outputs to identify significant differences in the temperature and precipitation predictions.

### AIM/CGE

We used an AIM/CGE model for this study, which is a recursive-type, dynamic, general equilibrium model that covers all regions of the world. Details of the model structure and mathematical formulas are provided by Fujimori *et al*. [27]. The main inputs were population, GDP, food preferences, and assumptions of energy technology and air pollution controls. The model provided energy consumption, agricultural and land use indicators, and emissions of GHGs and APs. AIM/CGE considered carbon dioxide, methane, nitrous oxide, and fluorine gas to be GHGs, while black carbon, carbon monoxide, ammonia, non-methane volatile organic compounds, mono-nitrogen oxides (NO_x_), organic carbon, and sulfur oxides (SO_x_) were treated as APs. The regional, geographical, and industrial classifications are shown in Table 1, Fig A, and Table A in S1 File, respectively.

**Table 1:** Regional classifications in the AIM/CGE.

Code	Region	Code	Region
JPN	Japan	TUR	Turkey
CHN	China	CAN	Canada
IND	India	USA	United States
XSE	Southeast Asia	BRA	Brazil
XSA	Rest of Asia	XLM	Rest of South America
XOC	Oceania	XME	Middle East
XE25	EU25	XNF	North Africa
XER	Rest of Europe	XAF	Rest of Africa
CIS	Former Soviet Union		

**Fig 2:**
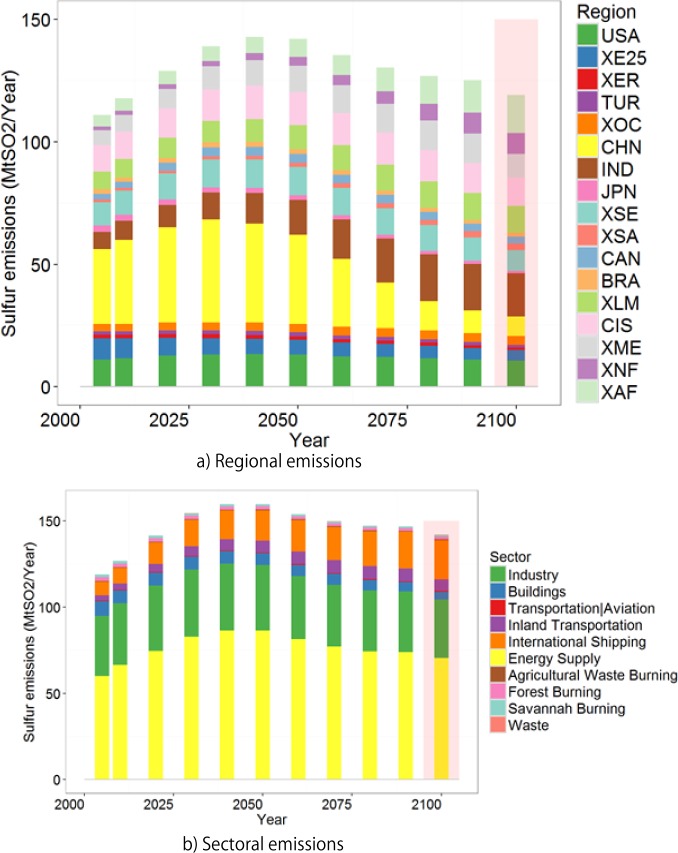
Global sulfur emissions by (a) region and (b) sector. The regional codes are defined in.

With respect to assumptions of the future, population and GDP were based on the Shared Socio-Economic Pathway 2 (SSP2) [28]. Among the SSPs, SSP2 is characterized as a middle-of-the-road scenario. In addition, assumptions related to energy technology, food, and land were based on the SSP2 [29, 30]. However, the original SSP2 sulfur emissions in the latter half of the 21^st^ century are low, owing to the assumption of continuous implementation of air pollution legislation [29, 30], and results in minimal sulfur effects on climate. Because we aimed to explore the effects of downscaling methods on sulfur emissions and obtain meaningful insights on the effects of downscaling on climate, we changed the sulfur emissions scenario from the original SSP2. Instead of large emissions throughout the 21^st^ century, we simply and arbitrarily assumed an annual 1% decrease in emissions coefficients. The projected sulfur emissions by region are shown in Fig 2.

### AIM/DS

#### The AIM/DS algorithm

AIM/DS is a tool used to downscale regionally aggregated emissions onto a grid-based map with a resolution of 0.5°. The algorithm is differentiated by sectors, and the sources of emissions are assigned to one of three groups (Table 2). In group 1, GDP or population are the drivers of the emissions. This algorithm is adopted mainly for energy-related emissions, which we assumed to have a relationship with GDP or population. Group 2 is downscaled in proportion to the total regional emissions. The base year map information is scaled up or down according to the total regional emissions. The base year represents the AIM/CGE simulation starting year (i.e., 2005). Therefore, the spatial distribution pattern for future scenarios is the same as that of the base year. Group 3 is downscaled in proportion to total global emissions with the base year spatial map. The basic logic in the case of group 3 is the same as that of group 2, but the logic is applied to cross-border sectors. It should be noted that land use-related emissions were classified as group 2 in this study, and the emissions of this sector would ideally consider changes in land use. However, because energy-related sectors are the main sulfur emitters, we used proportional downscaling. In future studies, we will likely follow an approach that includes information on changes in land use.

**Table 2:** Downscaling algorithm emission source groups and weight used.

Sectors	Group	Weight
Energy	1	GDP
Industry	1	GDP
Inland transport	1	GDP
Building	1	Population
Solvent	1	GDP
Waste	1	Population
Agriculture	2	
Agricultural waste	2	
Land use change	2	
Savanna burning	2	
International navigation	3	
Aviation	3	

Here, we explain the methodology of group 1 (Eqs 1–3), which is related to energy, solvent, and waste sector emissions, because the methods for group 2 and 3 are simple. We considered two factors, convergence and inertia. The basic logic was based on van Vuuren *et al*. [10], who assumed that the intensity of emissions converged among countries that belonged to the same aggregated region. Inertia can be interpreted as the level of technology, level of legislation, or sector structures that exist in the base year. Eq (1) represents these two factors.

$${EMG}_{i,t}=\sum_{r\in RI}\alpha_{t}∙{EI}_{r,t}∙{DV}_{i,t}+(1-\alpha_{t})∙{EMG}_{i,t-1}$$ (1)

where *EMG*_*i*,*t*_ is emissions in grid *i* and year *t*; *EI*_*r*,*t*_ is the emissions intensity in country *r* and year *t*; and *DV*_*r*,*t*_ is the driver in country *r* and year *t* (e.g., population and GDP). The factors α and (1- α) are the weighting coefficients between the convergence and inertia factors; α ranges from 0 to 1. If the factor is thought to be stronger than the inertia factor, the weight should be close to 1. A strong convergence factor indicates that the emissions intensity (e.g., emissions per GDP) of each grid cell converges to a certain value belonging to a region (e.g., the emissions intensity of a cell that belongs to Indonesia converges to the average of Southeast Asia). Conversely, a strong inertia factor indicates that the heterogeneity in the base year’s emissions intensity strongly remains (e.g., the emissions intensity of a cell that belongs to Indonesia remains the same as the base year intensity difference from the average of Southeast Asia). α is used to differentiate between the two methods. *RI* is a mapping from a set of country *r* to grid *i*. *DV*_*r*,*t*_ is an exogenous parameter, and *EMG*_*i*,*t-1*_ is the emissions in the previous year.

The parameter *EI*_*r*,*t*_ is the product of two terms, as shown in Eq (2). The former is the country-specific emissions intensity associated with the average emissions intensity change ratio in the aggregated region, and the latter is the emissions intensity in the aggregated region.

$${EI}_{r,t}={\left({EI}_{r,t0}∙\frac{\sum_{(r,rag)\in RM}{{EIAG}_{rag,t}}^{}}{\sum_{(r,rag)\in RM}{{EIAG}_{rag,t0}}^{}}\right)}^{\beta_{t}}∙\sum_{(r,rag)\in RM}{{EIAG}_{rag,t}}^{\left(1-\beta_{t}\right)}$$ (2)

where *EIAG*_*rag*,*t*_ is the average emissions intensity of the aggregated region *rag* (AIM/CGE; 17 regions) in year *t*, β is a parameter that represents the convergence of each region, *RM* is a mapping for the AIM/CGE-aggregated region to which the country belongs, and *t*0 is the base year (2005). If β = 0, all countries that belong to an aggregated region converge to the regional average intensity. We assumed that β was 1 in 2100 and 0 in the base year. The intermediate periods were connected linearly.

Aggregating the emissions from each country obtained by the direct solution of Eq (1) into the original 17 AIM/CGE regions yielded inconsistent numbers for the aggregated regional totals. Therefore, the *EMG*_*i*,*t*_ was updated by scaling using Eq (3).

$${EMG}_{i,t}^{*}=\sum_{r\in RI}{EM}_{r,t}∙\frac{{EMG}_{i,t}}{\sum_{r\in RI}{EMG}_{ii,t}}$$ (3)

where EMG^*^_i,*t*_ is the updated emissions in grid *i* and year *t*, and *EM*_*r*,*t*_ is the emissions in aggregated region *r* and year *t*.

#### Tests of the two methods for determining the parameter α

As shown in Eq (1), the weight coefficient α changes the pattern of emissions allocation. We examined two methods of determining α, M1-CONV and M2-INER. The basic strategy differentiating these two methods followed two extreme cases, as stated in the introduction. We used van Vuuren *et al*. [10] as the starting point, as no other reports on downscaling methodologies for global-scale emissions were available. van Vuuren *et al*. [10] assumed full convergence, which was an extreme case. The opposite extreme of this case would be strong inertia. If we could have found or created other extreme methods, we would have adopted them. However, to the best of our knowledge, these are the best available cases.

Method M1-CONV assumed that the intensity of emissions eventually converged to the aggregated regional average. α was initially 0.0 in 2005 and increased linearly to 1.0 in 2100. In this study, the GDP and population of each country were provided exogenously, and the behavior of GDP per capita differed from that assumed by van Vuuren *et al*. [10], who assumed that GDP per capita converged.

The M2-INER method assumed that a mixture of the convergence and inertia factors determined the intensity of emissions, and that inertia was more important than convergence. α was assumed to be 0.1 in 2100. We selected 0.1 arbitrarily as a sufficiently small value to observe the impact of the assumption of α. Ideally, it would be allowed to vary as a function of future socioeconomic assumptions. For example, if a scenario represented an SSP4-like world, characterized by inequality [31], α would be low. By contrast, if a scenario represented an SSP1-like world, characterized by equality and sustainability [31], α would be high.

### Population and GDP spatial allocation

We used national-level data for populations, urbanization rates, and GDP to generate the spatially gridded populations and GDPs. The base was the 2.5-arc-minute data from the Gridded Population of the World [32]. We used 0.5-arc-minute population data to produce population distributions within the 2.5-arc-minute grid cells as the initial values. Initial population data and national urban population data were used in urban areas. We set thresholds for population density based on the initial populations in the 0.5-arc-minute data so as to match national urban populations. We treated the 0.5-arc-minute grid cells above the threshold as urban cells and those below the threshold as rural cells. For the 30-arc-minute grid cells, we used urban population/area ratios as the urban index. The Greenhouse Gas Initiative database provided by the International Institute for Applied Systems Analysis [33] was the source of these data.

We used the rank-size rule to estimate the populations of urban grid cells. It is an empirical law used to estimate previous city populations [34], but is also applicable to future populations. Then the GDP distributions were basically allocated based on the populations. Several geographical constraints were considered, including mountains, water bodies, and urban sprawl. Supporting Information 2 provides additional details on the methodology.

### Configurations of the atmosphere–ocean GCM experiments

We used the coupled atmosphere–ocean global climate model MIROC5.2 in this study. MIROC5.2 is a minor upgrade of MIROC5.0 [24], and is modified as follows. The horizontal resolution of the atmosphere component uses a T42 spectral truncation (~2.56°). There are 40 vertical levels, and the top of the atmosphere is located at the 3-hPa pressure level, as in MIROC5.0. The simulation of solar radiation is improved in MIROC5.2 to deal with the infrared wavelength band 4–100 μm. In addition, the Minimal Advanced Treatments of Surface Interaction and Run-off (MATSIRO) land surface model [35], a parameterization for sub-grid scale snow cover distribution (SSNOWD) [36] and representation of wetlands, which store some snow meltwater [37, 38], is a new addition. MIROC5.2 uses COCO ver. 4.9 as the global ocean component [39], which includes several upgrades compared to MIROC5.0. A tri-polar grid is introduced in MIROC5.2 in place of the bi-polar generalized curvilinear coordinate used in MIROC5.0 [40]. The coordinate system is composed of a spherical coordinate portion south of 63.3°N and a bi-polar coordinate system in the Arctic region north of 63.3°N. The longitudinal grid spacing is 1° in the spherical portion. The latitudinal grid spacing is 1°cos*θ*, where *θ* is the latitude, while it is about 0.5° in the equatorial region and the Southern Ocean. The two coordinate singularities in the bipolar portion are placed at 63.3°N, 60°E in Greenland and 63.3°N, 120°W in Siberia. The zonal and meridional grid spacings are about 60 km and 33 km around the central Arctic Ocean, respectively. The number of vertical levels has been increased from 49 in MIROC5.0 to 62 in MIROC5.2. The profile of background vertical diffusivity has been changed in MIROC5.2. The empirical profile of Tsujino *et al*. [41], used in MIROC5.0, is used in MIROC5.2 below a depth of 50 m. However, above 50 m, the vertical diffusivity is set to 1.0×10^−6^ m^2^ s^-1^ in the uppermost 29 m and gradually increases to 1.0×10^−5^ m^2^ s^-1^ at 50 m. The turbulent mixing process in the surface mixed layer is also updated in MIROC5.2. Areas where the surface is covered with sea ice are assumed to experience no surface wave breaking, resulting in near-surface mixing. A combination of the smaller background vertical diffusivity above 50 m and the suppression of turbulent mixing under sea ice cover better represents surface stratification in the Arctic Ocean [42].

An online aerosol module of MIROC5.2, the Spectral Radiation-Transport Model for Aerosol Species (SPRINTARS) [43, 44], computes mass mixing ratios of the main tropospheric aerosols from emissions of aerosols and the aerosol precursors sulfate, carbonaceous aerosols (i.e., black carbon and organic matter), soil dust, and sea salt aerosols. The aerosol transport processes include emission, advection, diffusion, chemistry, wet deposition, dry deposition, and gravitational settling. SPRINTARS is coupled with radiation and cloud microphysics schemes to calculate the direct, semi-direct, and first and second indirect effects of aerosols. Because we compared the two experiments with different emissions of sulfur dioxide (SO_2_), a precursor of sulfate aerosols, only sulfate aerosols are described. Sulfate aerosols are formed mainly by chemical reactions of SO_2_ and dimethyl sulfide with oxides (hydroxide, ozone, and hydrogen peroxide), with monthly mean fields prescribed by the global chemical model, CHASER [45]. SPRINTARS explicitly treats the chemical reactions related to sulfate aerosols and SO_2_ dissolution into water. In addition, the SO_2_ dissolution process is applied to in-cloud scavenging of SO_2_. In the radiative calculation, the mode radii of the lognormal size distribution dependent on the relative humidity are assumed. The hygroscopic growth of sulfate aerosol particles is applied according to Tang and Munkelwitz [46]. The assumed volume-weighted refractive indices are used for the internal mixtures between aerosol particles and water. External mixing is assumed for sulfate, soil dust, and sea salt. More detailed processes on sulfate aerosols and other aerosols can be found in Takemura *et al*. [44].

We performed two experiments using MIROC5.2. Each experiment was driven by one of two SO_2_ emission datasets provided by the AIM/CGE. The emissions data for SO_2_ in the year 2100 were used in the experiments M1-CONV and M2-INER. For the other emissions data (e.g., black carbon and organic carbon) and forcing data (e.g., solar constant and volcanic aerosols), we applied pre-industrial conditions from 1850 in both experiments in which the anthropogenic emissions were zero. Both experiments were integrated for 40 years, and the last 30 years of data were analyzed to evaluate climatological differences between the M1-CONV and M2-INER methods that arose from difference in the SO_2_ emissions data. It should be noted that the distributions and radiative effects of sulfate aerosol in these experiments might have differed from that of future projections with varying GHGs and aerosol emissions, because background concentrations of other pollutants and changes in climate could have affected sulfate aerosols. To test the statistical significance of the difference between these two experiments, we used a 250-year preindustrial control simulation of MIROC5.2 (piControl).

## Results

### Overview of sulfur emissions

Fig B, C, D and F in S1 File show the global and regional populations, GDPs, and primary energy supplies, the main drivers of sulfur emissions. Fig 2 shows the global sulfur emissions computed by AIM/CGE by region and sector. Total sulfur emissions gradually increased from 112 Mt/year in 2005, peaked in roughly 2050 (158 Mt/year), and then declined to 140 Mt/year in 2100. Because emissions were computed with driving forces (mainly energy consumption) and emissions coefficients, the decline only occurred in the latter period. These emissions were obviously higher than any of the RCPs shown by van Vuuren *et al*. [2] and slightly higher than the maximum level in the Intergovernmental Panel on Climate Change Fifth Assessment Report (IPCC AR5) scenarios (Riahi *et al*. [47]), implying that the scenario used in this study was near the maximum possible emissions. The main reason for these high emissions was the conservative air pollutant legislation assumption adopted in this study. A regional breakdown showed that China was the largest emitter in 2005, but its emissions became much lower by 2100. Meanwhile, India increased its total amount and proportional share of sulfur emissions. In addition, sulfur emissions increased in Africa. Both India and Africa were projected to experience steady increases in income and energy consumption over time (Figs B and D in S1 File). A breakdown by sectors indicated that energy-related emissions, such as emissions from the industrial and energy sectors, accounted for most of the total sulfur emissions, and this characteristic remained throughout the 21^st^ century. Emissions from the energy sector decreased over time, industrial emissions remained more or less constant, and transportation-related emissions increased. As a result, the share of emissions from the energy sector declined from 54% in 2005 to 43% in 2100, whereas the industrial and transportation sectors contributions to sulfur emissions increased from 31% to 38% and from 3% to 8%, respectively, in the same timeframe.

### Spatial distribution of sulfur emissions

**Fig 3:**
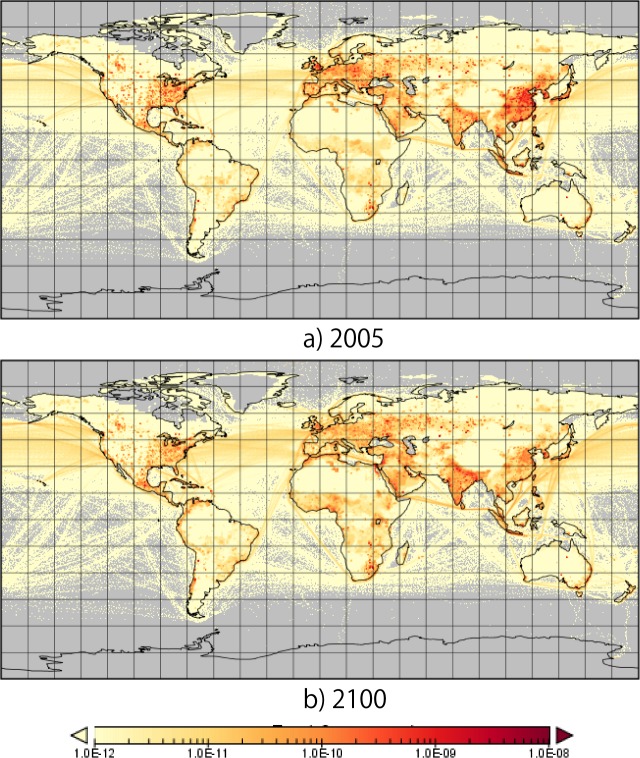
Spatial distribution of sulfur emissions (Kg/m^2^/sec) in 2005 and 2100 in M1-CONV (sector total).

Fig 3 shows the spatial distribution of sulfur emissions in 2005 and 2100 projected by M1-CONV (the 2050 maps are presented in Fig F and G in S1 File). In 2005, there were high emissions from China, the east coast of the United States, and Europe. The distribution of emissions differed in 2100. Emissions from southern Asia, particularly the Himalayan region (i.e., Bhutan and Nepal), were remarkably high in 2100. Second, high emissions were observed in the west coast of Africa (e.g., Cote d’Ivoire) and eastern central Africa (e.g., Kenya). These trends reflected the projected increases in income and energy consumption in Africa and southern Asia. Third, emissions from China were projected to decline from 2005 to 2100, particularly along the east coast of China.

**Fig 4:**
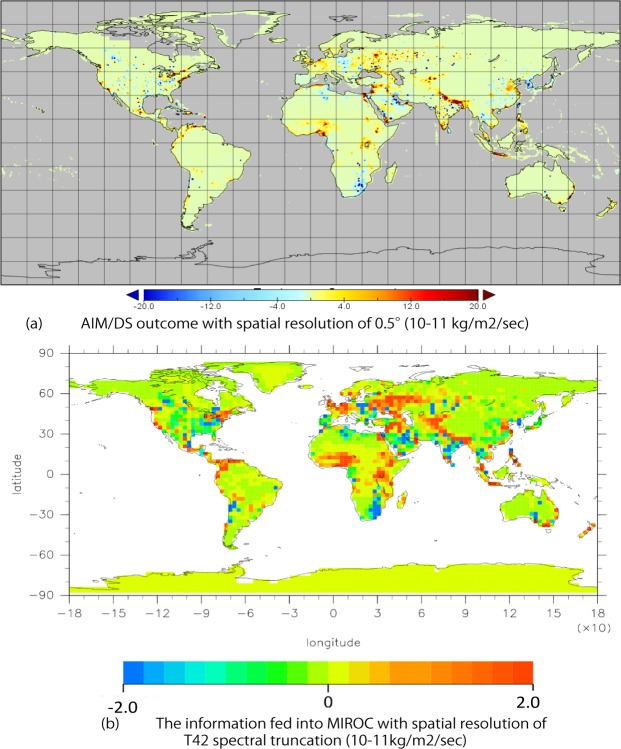
Spatial difference in sulfate emissions (10^−11^ kg/m^2^/sec) between M1-CONV and M2-INER. Difference between the two methods (a) at a 0.5° grid resolution computed by AIM/DS and (b) using T42 spectral truncation (~2.56°) resolution in MIROC.

**Fig 5:**
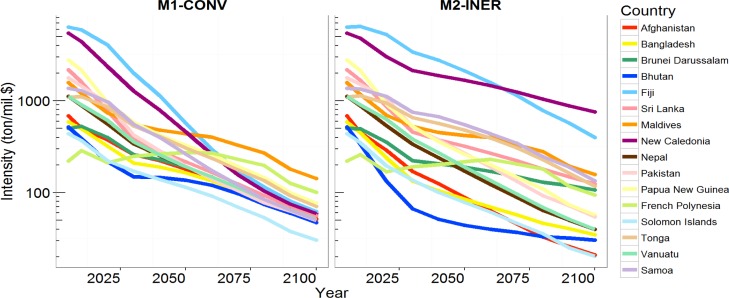
Emissions intensities (emissions per GDP) for countries in the Rest of Asia region based on M1-CONV and M2-INER.

Fig 4 illustrates the difference between M1-CONV and M2-INER computed by AIM/DS. The red shading indicates grid cells in which the M2-INER projections are smaller emissions than those of M1-CONV, while the blue shading indicates the opposite. Red-shaded regions include Nepal, western Russia, eastern United States, sub-Saharan Africa, North Africa, and the Middle East. Blue-shaded regions include southeastern Africa, Eastern Europe, the Korean Peninsula, the center of North America, and South Africa. These differences reflected the strength of the convergence effect. However, it was not straightforward to determine why the strength of the convergence effect differed. In principle, the reason was that the density of emissions (emissions per area) was initially higher in some regions than in other regions, resulting in remarkably large differences, although the ratio of the two methods was not particularly large for those regions. For example, in the Rest of Asia region (i.e., Bhutan and Nepal), M1-CONV projected higher emissions than M2-INER. Fig 5 illustrates the emissions intensities for the Rest of Asia region by country. The M1-CONV method did not converge because of the inclusion of non-energy sectors, which were not constrained to converge. The emissions converged more strongly in the latter half of the 21^st^ century for M1-CONV than for M2-INER. The intensities of emissions from Bhutan and Nepal were relatively low in 2005. In the M1-CONV method, the proportion of emissions from these countries increased, converging toward the average of this region, which was higher than the emissions from Bhutan and Nepal. In the M2-INER method, the emissions from Bhutan and Nepal remained unchanged from the base year. This revealed that these two countries did not always have a high intensity of emissions in the Rest of Asia region, and the level of the intensity did not entirely explain the spatial distribution in the maps. Instead, emissions per unit area were the primary driver. Another example was Egypt, which had a high emissions density in a very limited area, the northeastern part of the country, resulting in the dark red shading of Egypt in Fig 4. As was the case for Bhutan and Nepal, Egypt did not always have the highest emissions intensity in North Africa, the region that included Egypt (Fig I in S1 File). The other factor of the different geographical allocations was the difference in GDP growth across countries within aggregated regions, which was observed in the sub-Saharan region. In the GDP scenario assumption, Nigeria had much higher growth than South Africa and higher emissions were formed in Nigeria (Fig J in S1 File).

Fig 4 illustrates the difference between the two methods in the T42 spectral truncation (~2.56°) resolution used in MIROC. Geographical features of the differences in emissions between M1-CONV and M2-INER in the original horizontal resolution were observed in the emissions differences in the MIROC5.2 resolution. Therefore, the geographical features of the emissions differences in the original horizontal resolution were appropriately implemented in the emissions data used in the MIROC5.2 experiments. In addition, by comparing 20-year averages of the mass column loading of sulfate aerosol in M1-CONV and M2-INER, significant differences in the mass column loading of sulfate aerosol were widely observed (Fig H in S1 File).

### Frequency distribution of emissions density and intensity

**Fig 6:**
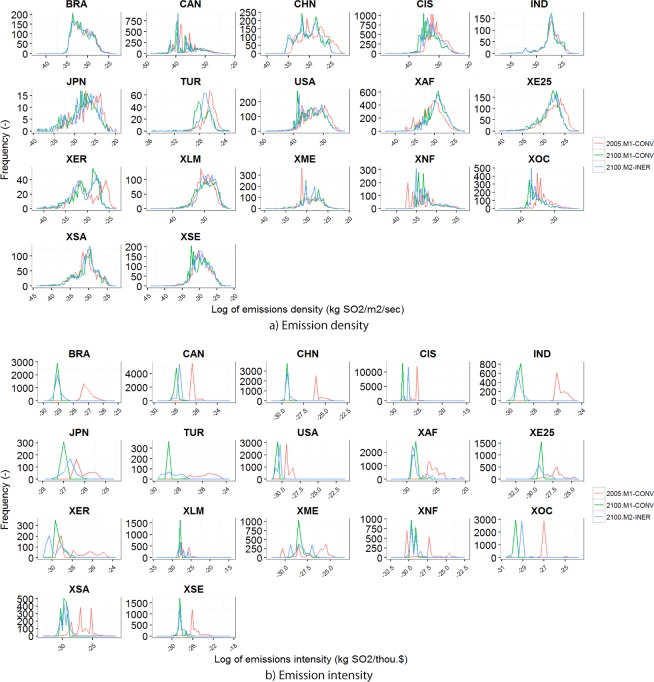
Frequency distribution of emissions density and intensity for the sector total. The emissions density represents emissions per area and the emissions intensity is emissions per GDP. The regional codes are defined in Table 1.

The frequency of distribution of emissions density and intensity can be investigated to better understand how spatial downscaling works. Fig 6 illustrates the frequency distribution of grids as a function of the emissions density (per area) and emissions intensity (per GDP) generated by the M1-CONV and M2-INER methods for the base year and 2100. It should be noted that the scale for the abscissa is logarithmic, and the ranges differ between regions. As the emissions intensity converged, the spatial allocation of emissions approached that of the GDP, and there was a large change in the emissions density distribution. The distributions for 2100 and 2005 simulated with M2-INER were similar. However, the M1-CONV distributions did not fully converge. There are two possible explanations for this behavior. First, the GDP density did not change substantially, and the emissions density could eventually become similar to the GDP density. Second, the scale of the abscissa is logarithmic, and the changes are difficult to discern. Meanwhile, similar figures made using emissions intensity based on emissions per GDP showed no apparent differences. The emissions intensities based on M1-CONV converged strongly, whereas those based on M2-INER did not.

Next, we analyzed the distribution changes based on statistical indicators using the SD and IQR of both emissions density (Table 3) and intensity (Table 4) in the energy sector, which was the sector that accounted for the greatest share of sulfur emissions. As already noted, the emissions density differed little between 2005 and 2100 (Table 3), and the difference between the emissions density projected by the M1-CONV and M2-INER methods was not very obvious. By contrast, the statistics relevant to emissions intensity (Table 4) differed drastically between M1-CONV and M2-INER. In the M1-CONV projection, the emissions intensity converged to 0 in 2100, which was not observed in the M2-INER projection, although the emissions intensities calculated in M2-INER decreased for most countries.

**Table 3:** Statistics for emissions density (kg SO_2_/m^2^). SD, standard deviation; IQR, interquartile range. The regional codes are defined in Table 1.

	SD			IQR		
	2005	2100		2005	2100	
		M1-CONV	M2-INER		M1-CONV	M2-INER
BRA	1.77	1.98	1.97	2.28	2.97	2.90
CAN	4.47	4.39	4.47	6.38	6.39	6.38
CHN	2.69	2.76	2.74	4.05	4.06	4.07
CIS	2.95	2.84	2.87	4.34	3.70	3.93
IND	1.94	1.91	1.93	1.48	1.59	1.62
JPN	2.91	3.15	2.97	3.80	4.08	3.90
TUR	2.36	1.12	1.25	3.07	1.58	1.92
USA	3.89	3.73	3.75	5.49	6.02	5.80
XAF	2.14	2.33	2.24	2.50	2.88	2.78
XE25	2.55	2.63	2.48	3.14	3.39	3.05
XER	2.30	2.68	2.42	2.55	3.79	3.16
XLM	3.11	3.61	3.18	3.89	5.51	4.56
XME	1.90	2.20	1.96	1.93	3.06	2.41
XNF	2.65	2.50	2.54	3.91	2.79	3.73
XOC	2.31	2.62	2.41	2.94	3.10	3.00
XSA	2.74	3.02	2.69	3.32	4.42	3.51
XSE	2.61	2.59	2.57	3.85	3.88	3.90

**Table 4:** Statistics for emissions intensity (kg SO_2_/US$GDP). SD, standard deviation; IQR, interquartile range. The regional codes are defined in Table 1.

	SD			IQR		
	2005	2100		2005	2100	
		M1-CONV	M2-INER		M1-CONV	M2-INER
BRA	1.26	0.00	0.60	0.21	0.00	0.08
CAN	1.03	0.00	0.79	0.00	0.00	0.00
CHN	0.61	0.00	0.48	0.01	0.00	0.01
CIS	1.02	0.00	0.53	0.00	0.00	0.00
IND	0.82	0.00	0.56	0.07	0.00	0.14
JPN	0.50	0.00	0.55	0.02	0.00	0.35
TUR	2.27	0.00	0.95	2.60	0.00	0.71
USA	1.68	0.00	1.18	0.24	0.00	0.21
XAF	1.08	0.00	0.66	0.81	0.00	0.36
XE25	1.13	0.00	0.84	1.68	0.00	0.95
XER	1.34	0.00	0.60	0.37	0.00	0.91
XLM	1.60	0.00	1.22	1.19	0.00	1.65
XME	1.24	0.00	1.12	0.91	0.00	0.86
XNF	1.59	0.00	1.37	2.01	0.00	1.37
XOC	0.73	0.00	0.61	0.00	0.00	0.00
XSA	1.67	0.00	1.04	2.60	0.00	1.21
XSE	1.08	0.00	0.71	0.10	0.00	0.29

### Climate variables

**Fig 7:**
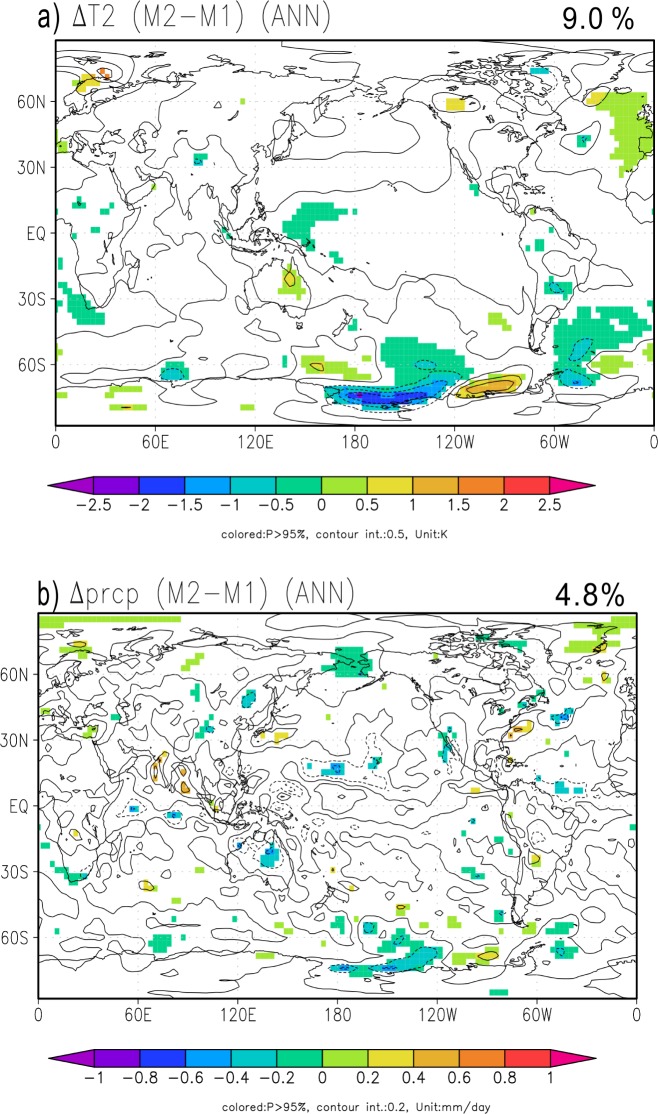
Annual mean (a) temperature and (b) precipitation differences between M1-CONV and M2-INER (M2-INER–M1-CONV). The shaded areas represent areas where the differences differed statistically based on a *t*-test (*p* ≤ 0.05). The percentage in the upper-right corner of the figure represents the proportion of surface area with significant difference to the global surface area.

The globally averaged 30-year mean temperature difference in the climate simulation between the M1-CONV and M2-INER methods (-0.0076 ± 0.053°C) was not significant. The confidence interval was estimated with a *t*-test at the 95% significance level. We assumed a degree of freedom of 18, which was estimated by considering the persistence in a first-order autoregression of global annual mean temperature time series of the piControl [48]. The spatial distributions of the differences in the annual mean temperature and precipitation between the two methods are shown in Fig 7 (seasonal differences are shown in Figs K and L in S1 File). The colored areas indicate differences that are statistically significant at the 95% level in *t*-tests with a degree of freedom of 58 (assuming 1 per year). Although there were local differences in the M1-CONV and M2-INER temperatures in 2100, the differences were small in most cases, and temperature differences significantly differed from 0 only in 9.0% of the total area. However, these significant differences may not represent clear evidence of sensitivity to the emission data, because 5% of the grid values are expected to be significant at the 95% significant levels by chance if the grids are independent of one another. Furthermore, in climate systems, considerable spatial correlations of the internal natural variability can lead to more or less than 5% of the total area with significant differences. To evaluate the possibility that these proportions of significant cases resulted from the internal natural variability alone, we applied a field significance test [26, 49, 50] using the outputs of the piControl run. We computed the differences between two non-overlapped 30-year period averages of the 250-year piControl, and estimated the area fractions of grids with significant differences. We repeated these processes for all 28 combinations of two non-overlapped 30-year periods, and found that 96% (27/28) of all combinations had area fractions of significant difference larger than 9.0% when M1-CONV was subtracted from M2-INER. Therefore, there was no clear evidence that the two methods caused apparent differences in the projections of temperature change.

Similarly, the differences in global mean precipitation were not significant (0.0022 ± 0.0054 mm day^-1^; degrees of freedom: 44). The differences were statistically significant in only 4.8% of the areas. In the field significance test, 93% of all of the combinations of two 30-year piControl segments (26/28) had areas with significant differences larger than 4.8%. Therefore, the 4.8% of the global area that exhibited significant differences occurred by chance due to internal natural variability.

## Discussion and Conclusions

We described two downscaling methods of the AIM modeling framework and compared the two methods using the same aggregated sulfur emissions. One method is based on the convergence of emissions intensity, and the other is based on inertia. We performed climate model simulations using MIROC with spatial distributions of sulfur emissions based on the two methods, and then investigated the climate responses. The spatial allocation of emissions differed between the two methods. This difference was obvious in the case of emissions intensity, but was much less apparent in the case of emissions density. The projected temperature and precipitation in 9.0% and 4.8% of the total global area, respectively, differed significantly between the two methods. However, the field significance tests showed that these could have been produced by natural internal variability alone. Therefore, there was no clear evidence that the differences between the two downscaling methods led to additional significant uncertainties in climate projections. The conclusion derived from this analysis is that the differences in the spatial allocations investigated in this study did not have a significant systematic impact on global or regional climate.

This study had several limitations. First, we only tested sulfur emissions; considering other gases could change the climate projections. Sulfur is a major gas that affects the pattern of cloud generation and radiative forcing, but black carbon might also be worth considering. We tested the downscaling method and determined the characteristics of the methods. Therefore, we limited the study to sulfur. However, it may be valuable to include other gases in future studies. Second, although the spatial allocation of GDP and population affect the geographical allocation of emissions, we used simple methods to allocate emissions. Using different spatial drivers could result in different emissions results, and the implications in terms of climate variability would then differ from those in this study. The M1-CONV method, for which the emissions intensity strongly converged, would be particularly sensitive to such changes. Third, this study focused entirely on climate responses, but it would have been beneficial to use the spatial data on emissions to study air pollution or its associated health effects. The conclusions may have been affected by the downscaling method, and more elaborate methodologies should be considered in future studies. Finally, we used a single climate model, MIROC, and conclusions of such analyses may depend on the climate model used. Thus, our results should be interpreted accordingly.

## Supporting Information

S1 File .pdf file includes 1) regional and sector classification of AIM/CGE, 2) description of population and GDP downscaling method, 3) main drivers of sulfur emissions and 4) supporting figures of results. (PDF)
