# Supplementary material for: Downscaling Global Emissions and Its Implications Derived from Climate Model Experiments
Source: PLoS One. 2017 Jan 11;12(1):e0169733. doi: 10.1371/journal.pone.0169733 (PMC5226776; doi:10.1371/journal.pone.0169733)
Supplement: S1 File — (PDF) [file pone.0169733.s001.pdf]

Supporting Information for

Global Emissions Downscaling and Its Implication derived from GCM experiments

Shinichiro Fujimori, Manabu Abe, Tsuguki Kinoshita, Kiyoshi Takahashi, Toshihiko Masui, Tomoko Hasegawa, Hiroaki Kawase, Hideo Shiogama, Hiroaki Tatebe

1. Classification of AIM/CGE 2
2. Population and GDP distribution Method 3
3. Main drivers of sulfur emissions 5
a) Population 6
b) GDP 6
4. Supporting figures for results 7

1. Classification of AIM/CGE

SI Table 1 Industrial classification

Agricultural sectors	Energy supply sectors	Other production sectors
Rice	Coal mining	Mineral mining and other quarrying
Wheat	Oil mining	Food products
Other grains	Gas mining	Textiles, apparel, and leather
Oil seed crops	Petroleum refinery	Wood products
Sugar crops	Coal transformation	Paper, paper products, and pulp
Other crops	Biomass transformation (1st generation)	Chemical, plastic, and rubber products
Ruminant livestock	Biomass transformation (2nd generation with energy crops)	Iron and steel
Raw milk	Biomass transformation (2nd generation with residues)	Nonferrous products
Other livestock and fisheries	Gas distribution	Other manufacturing
Forestry	Coal-fired power	Construction
	Oil-fired power	Transport and communications
	Gas-fired power	Other service sectors
	Nuclear power	CCS service
	Hydroelectric power	
	Geothermal power	
	Photovoltaic power	
	Wind power	
	Waste biomass power	
	Other renewable energy power generation	
	Advanced biomass-power generation	

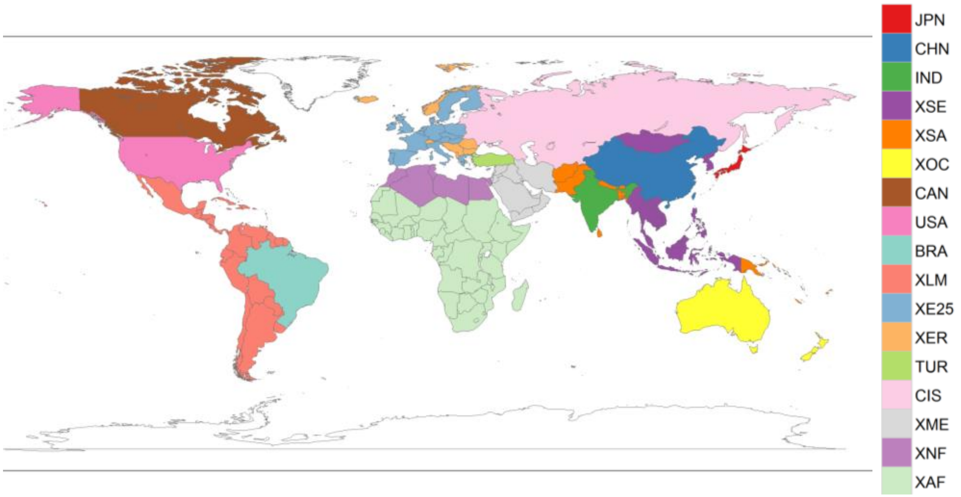

Fig S 1 Geographical model region in AIM/CGE.

2. Population and GDP distribution Method

As explained in the methodological section, we used the rank-size rule to make the spatially explicit population map. The rank-size rule is expressed by Equation (1).

$$P_i = b \cdot R_j^a \qquad (1)$$

where P denotes a city population and R denotes rank order, while a and b are constants. Population of rural grid cells are estimated in two case. If a grid cell had a population density (ϕ) above a certain value (Φ), the following was applied.

$$p_i^{x+1} = k_1 p_i^x \qquad (2)$$

where k_1 is the rate of urban population increase. The constant (Φ) is the threshold population density for urban areas for that country in the year 2000. p_i^x is the population of grid cell *i* at time *x*. The population change in a grid cell equal to or below the threshold Φ was handled as in Equation (3).

$$p_i^{x+1} = k_2 p_i^x = \left[k_1 + b\left(\log\phi_i - \log\Phi\right)\right]p_i^x \qquad (3)$$

where k_2 denotes the rate of population increase of the rural grid cell, and *b* is a constant that satisfies the following equation.

$$P_{rural}^{x+1} = \sum_{i=1}^{N_{rural}} p_i^{x+1} = \sum_{i=1}^{N_{rural}} \left[k_1 + b\left(\log\phi_i - \log\Phi\right)\right]p_i^x \qquad (4)$$

The expansion of cities must not be disregarded when making a projection up to the year 2100. Accordingly, we represented the expansion of cities by the following method. It has also been shown that the rank-size rule stands for not only the population of cities (1), but areas of cities (2). The urban area in each country has a high correlation with GDP and population. Therefore, if the sizes of cities are assumed to expand in accordance with the same rank-size rule as in Equation (1), city sizes in respective grid cells will expand by matching proportions. Even though we assumed this relationship between city sizes and GDP, in practice the expansion of city sizes is frequently subject to geographical constraints. Therefore, we applied geographical constraints to the expansion of city sizes. In making the urban/rural judgment for 0.5-arc-minute grid cells, of rural cells that are adjacent to urban cells, we treated cells that are land and have a slope of 5º or less as semi-urban cells. In expansion of a city, the semi-urban cells were converted to urban in sequence, starting from the grid cells with the highest population densities. A change in city size in this case is provided by Equation (5).

$$\frac{{}^{x+1}A_i}{{}^{x}A_i} = \frac{{}^{x+1}GDP}{{}^{x}GDP}\frac{\sum {}^{x}a_{semi-urban}}{\sum {}^{x}a_{neighbor}} \qquad (5)$$

where $a_{semi-urban}$ denotes the area of semi-urban cells, and $a_{neighbor}$ denotes the area of all cells adjacent to urban cells. Therefore, increases in size of cities that are surrounded by mountains or sea were small.

Urban sprawl here represents expansion in city sizes within the 30-arc-minute grid cells. However, there are some cities that straddle two or more of these cells. In a megacity in particular, there may be a 30-arc-minute grid cell that is wholly within the city. The size of the city does not expand within this 30-arc-minute grid cell, but the size of the city does expand in surrounding grid cells. Equation (6) expresses the contribution of a grid cell i to the increase in city size in grid cell $i+1$.

$$\Delta^{x+1}A_{i+1,i} = {}^{x}A_i \frac{{}^{x+1}GDP}{{}^{x}GDP} \frac{\sum {}^{x}a_{edge\_i,i+1}}{\sum {}^{x}a_{neighbor}} \quad (6)$$

where $\Delta^{x+1}A_{i+1,i}$ denotes the increase in size in grid cell $i+1$ that is caused by grid cell i. $a_{edge\_i,i+1}$ is the area of 0.5-arc-minute grid cells, among the 0.5-arc-minute grid cells within grid cell $i+1$, that are adjacent to grid cell i and at which both the 0.5-arc-minute grid cells in grid cell $i+1$ and the adjacent 0.5-arc-minute grid cells in grid cell i are urban cells.

3. Main drivers of sulfur emissions

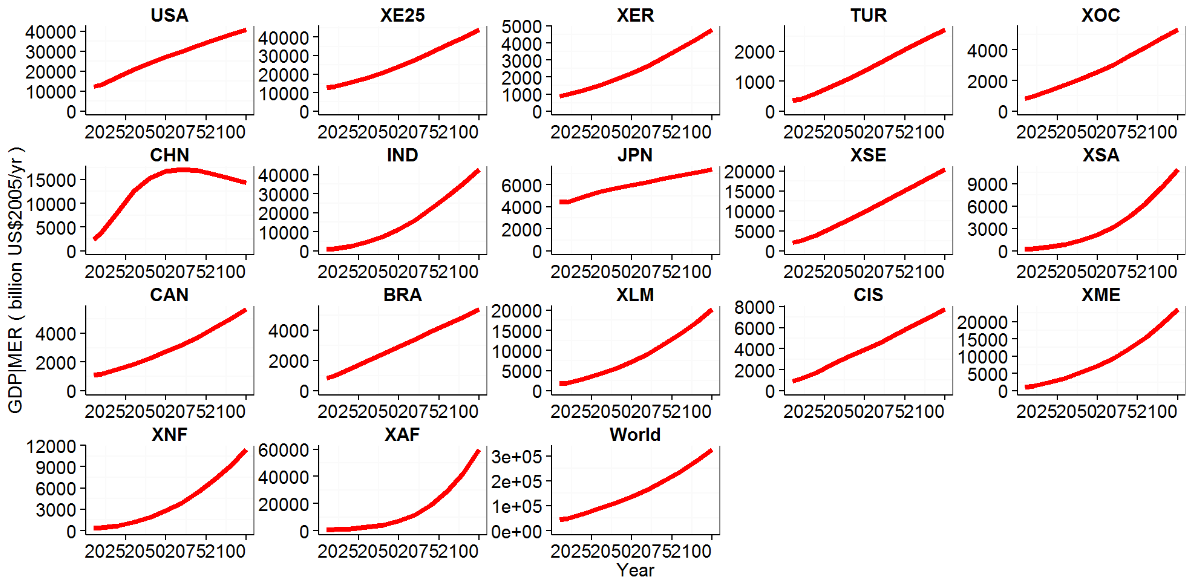

Fig S 2 GDP

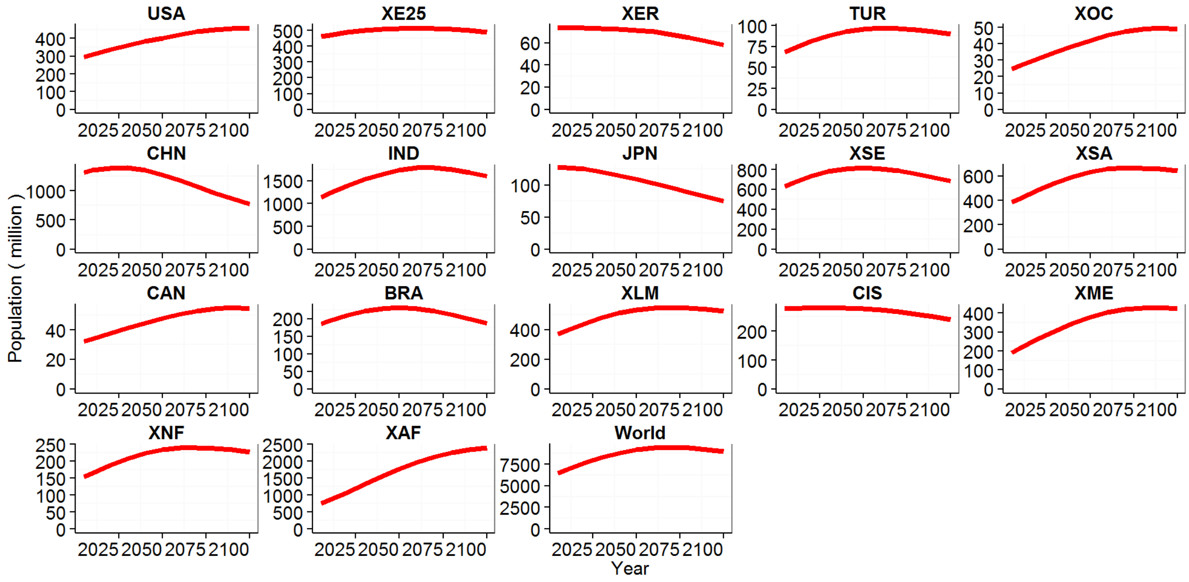

Fig S 3 Population

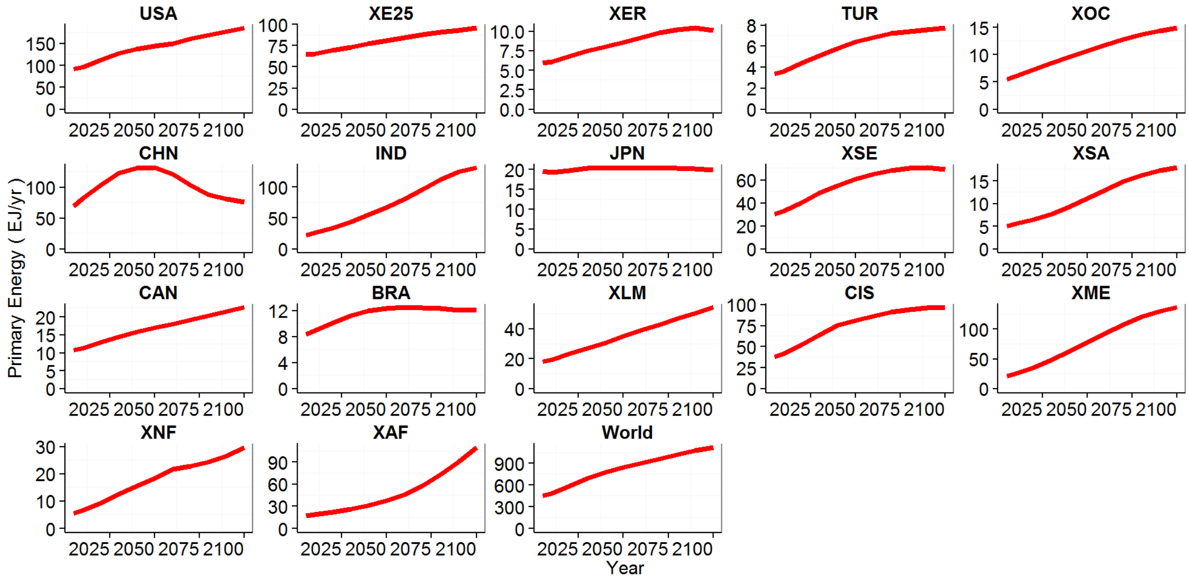

Fig S 4 Primary energy

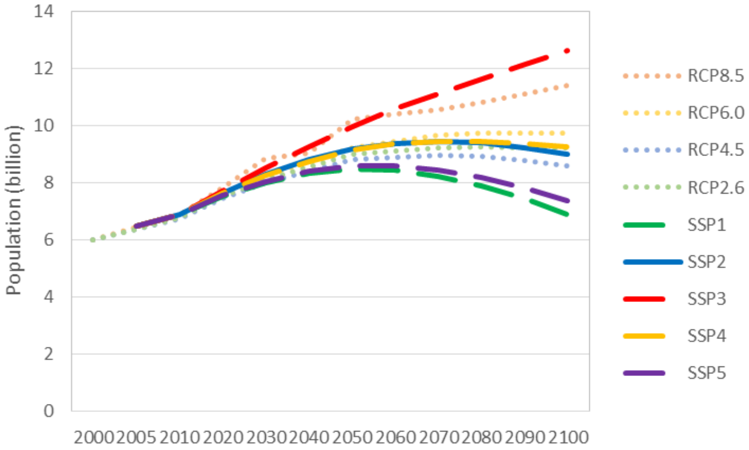

a) Population

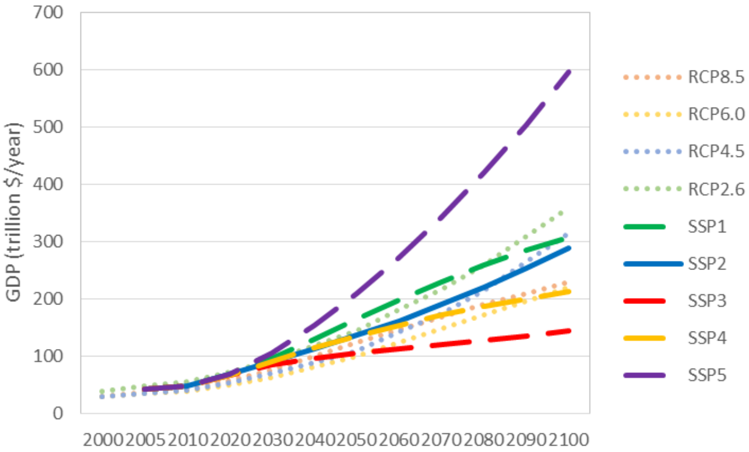

b) GDP

Fig S 5 GDP (a) and population (b) scenarios of RCPs and SSPs

4. Supporting figures for results

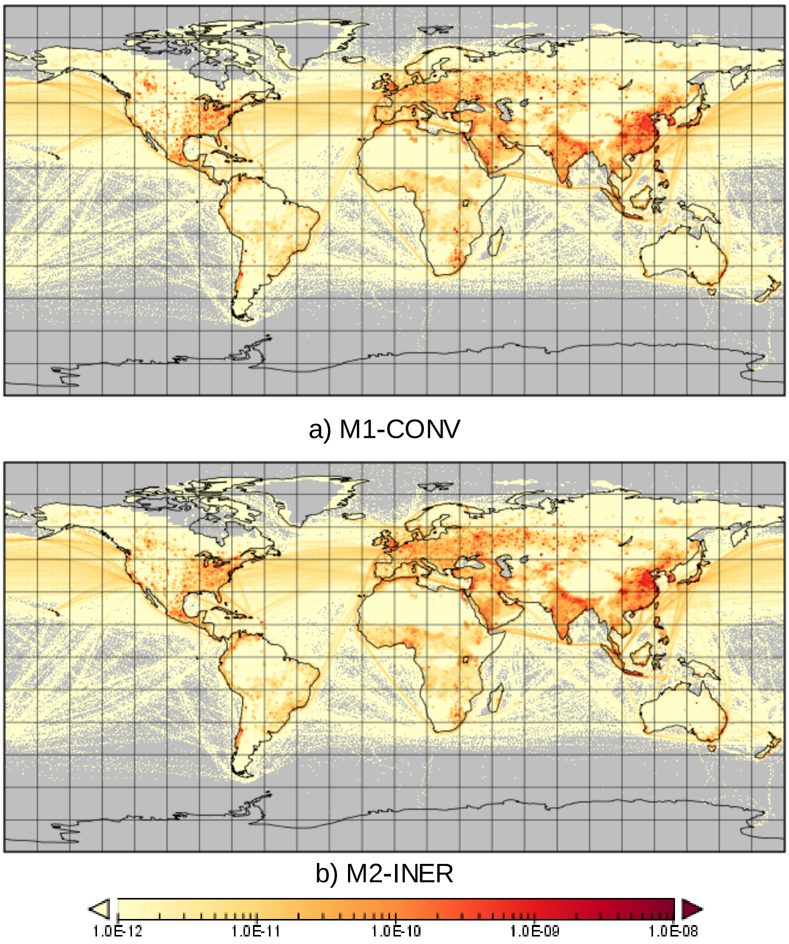

Fig S 6 Spatial distribution of sulfur emissions in 2050 with M1-CONV and M2-INER (sector total). Unit is Kg/m^2/sec

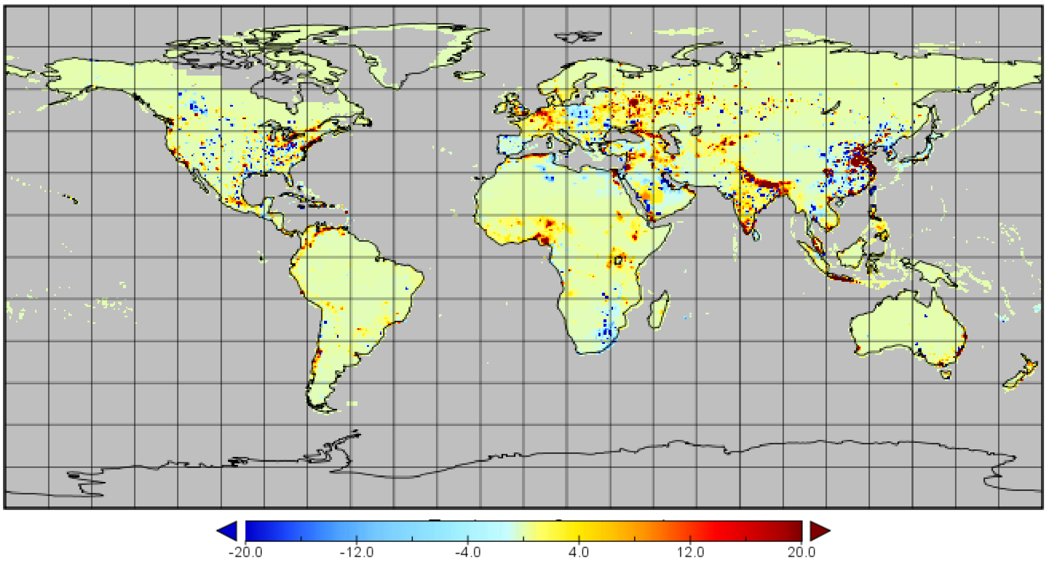

Fig S 7 Spatial sulfate emissions differences between M1-CONV and M2-INER in 2050. The two methods differences in 0.5° grid resolution which is computed by AIM/DS and the unit is 10^{-11}kg/m^{2}/sec.

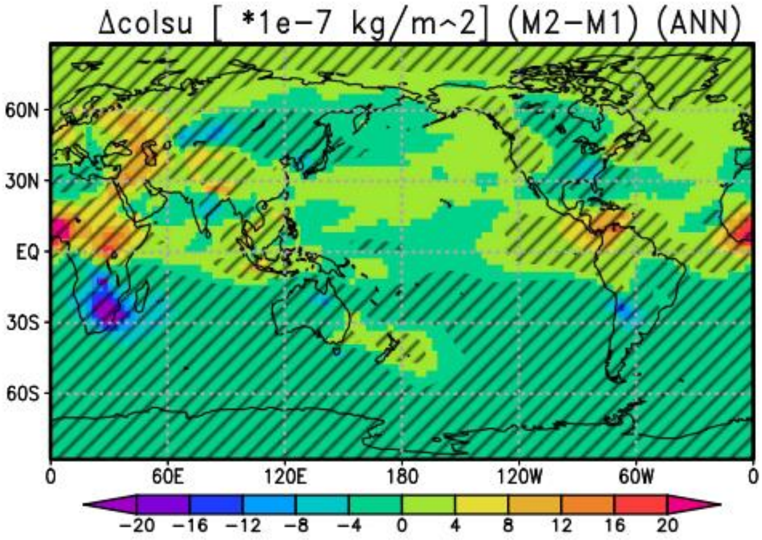

Fig S 8 Differences in 20-yr averages of the mass column loading of sulfate aerosol between M2-INER and M1-CONV. Unit is 1e-7 kg m^{-2}. Hatching indicate grids where differences are statistically significant at 95% confidence level.

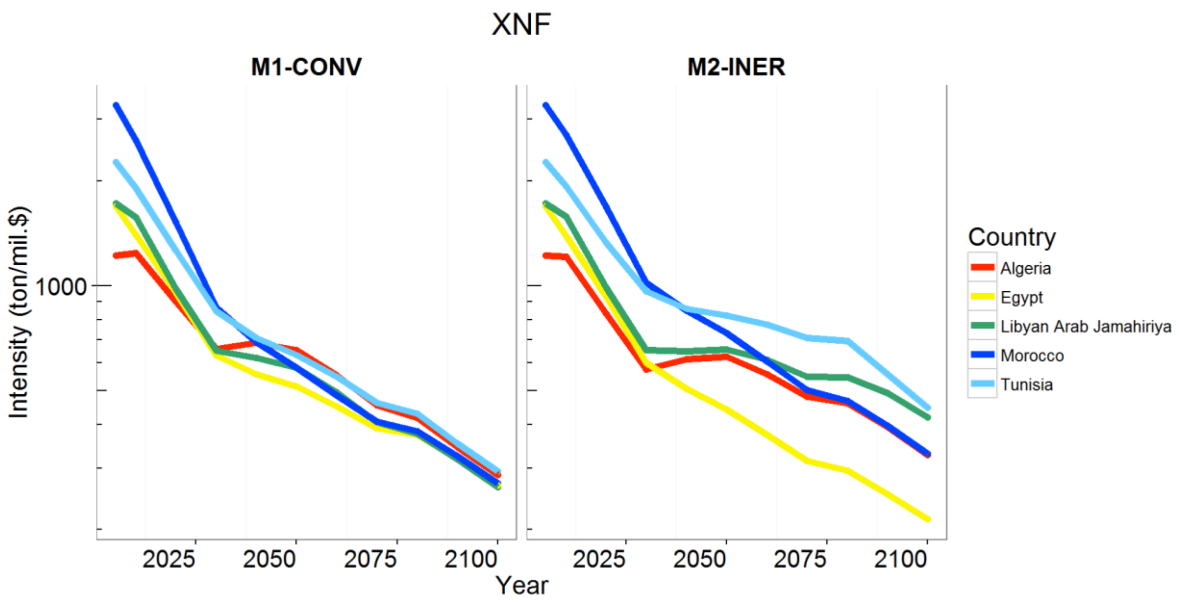

Fig S 9 Emissions intensity (emissions per GDP) across countries in North Africa generated by M1-CONV and M2-INER (sector total)

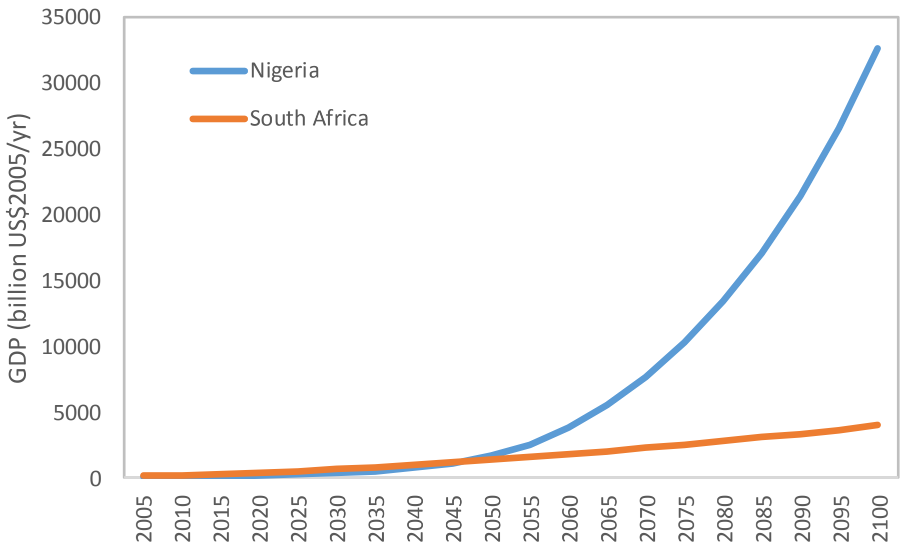

Fig S 10 GDP assumption in Nigeria and South Africa

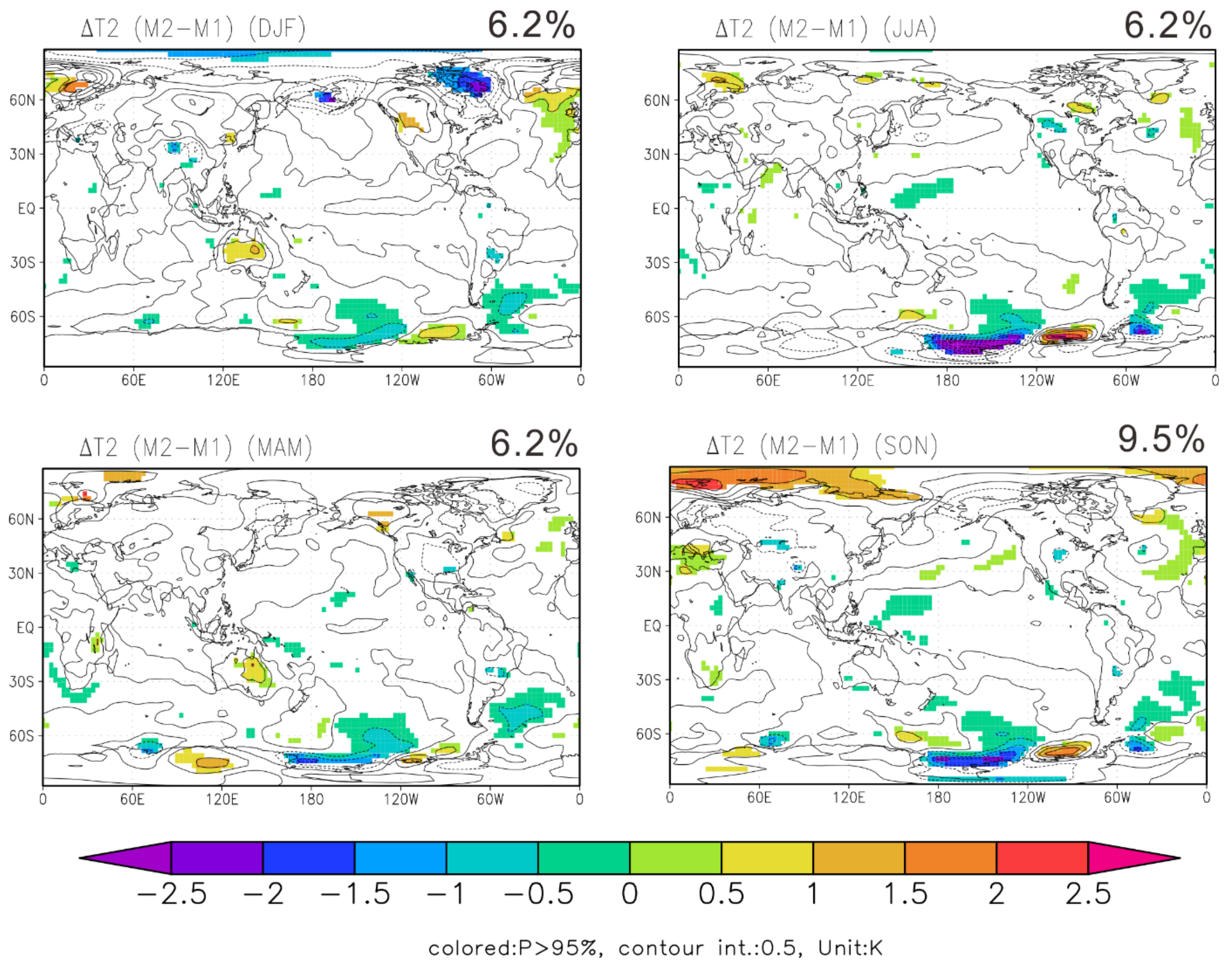

Fig S 11 Seasonal temperature differences between M1 and M2 (M2-M1). The four figures are December to February (DJF), March to May (MAM), June to August (JJA) and September to November (SON). The colored area represents statistically significant by t-test (95%). The % value in the upper-right of the figure means ratio of surface area with the significant difference to global surface area.

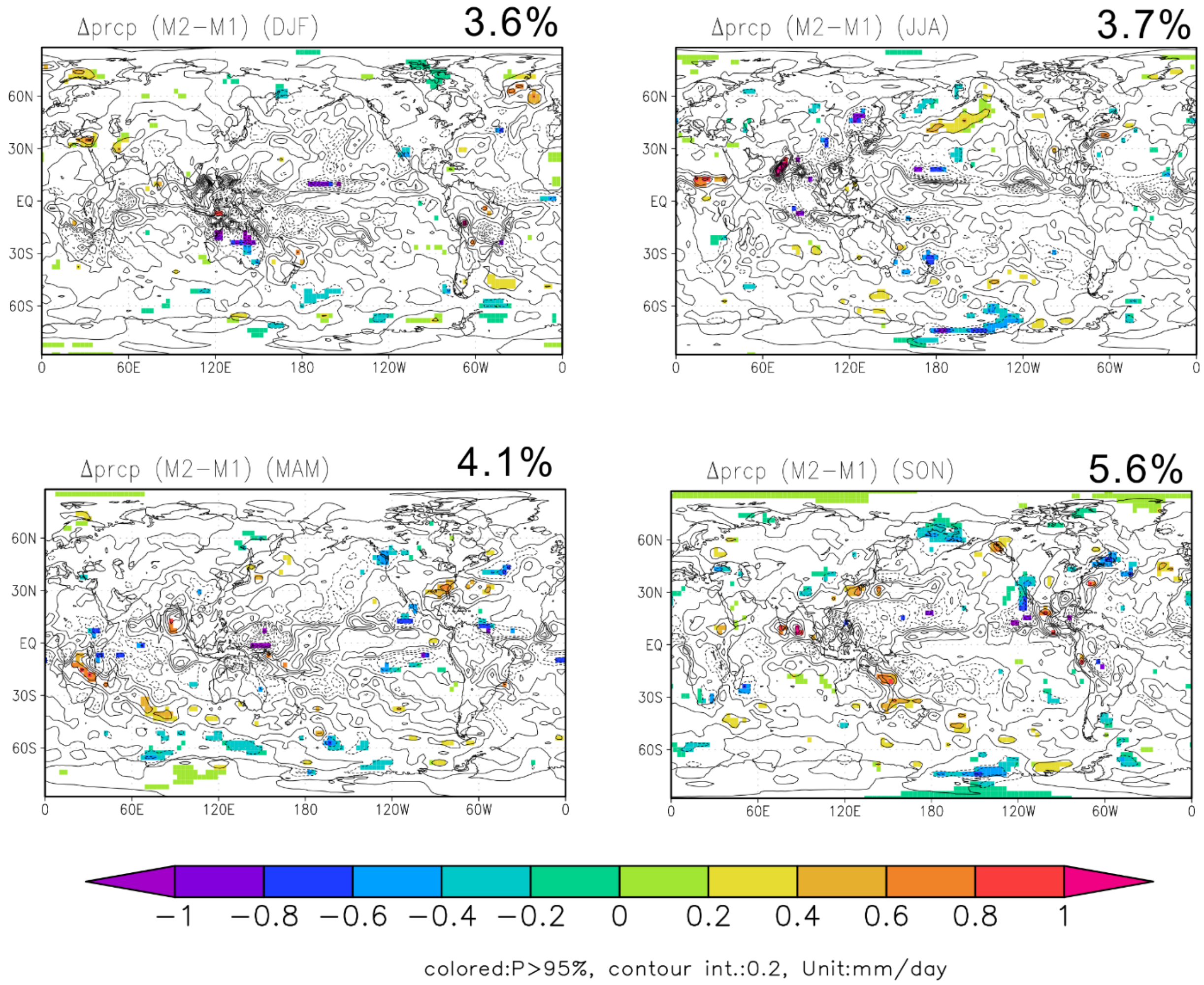

Fig S 12 Seasonal precipitation differences between M1 and M2 (M2-M1). The four figures are December to February (DJF), March to May (MAM), June to August (JJA) and September to November (SON). The colored area represents statistically significant by t-test (95%)

References

1. Nitsch V. Zipf zipped. Journal of Urban Economics. 2005;57(1):86-100.

2. Kinoshita T, Kato E, Iwao K, Yamagata Y. Investigating the rank‐size relationship of urban areas using land cover maps. Geophysical Research Letters. 2008;35(17).
